# Factors Influencing Primary Care Physicians’ Intent to Refer Patients With Hypertension to a Digital Remote Blood Pressure Monitoring Program: Mixed Methods Study

**DOI:** 10.2196/64933

**Published:** 2025-03-24

**Authors:** Jennifer J Wu, Ross Graham, Julie Çelebi, Kevin Fraser, Geneen T Gin, Laurel Dang, Esmatullah Hatamy, Amanda Walker, Courtney Barbato, Ottar Lunde, Lisa Coles, Parag Agnihotri, Cassandra Morn, Ming Tai-Seale

**Affiliations:** 1 Department of Family Medicine University of California, San Diego San Diego, CA United States; 2 Department of Sociology University of California, San Diego San Diego, CA United States; 3 Department of Medicine University of California, San Diego San Diego, CA United States; 4 Community Care University of California, San Diego San Diego, CA United States

**Keywords:** digital health, primary care, electronic health records, referral, hypertension, remote monitoring, remote blood pressure, digital technology, mobile phone, mixed method, quantitative analysis, linear regression, clinical information

## Abstract

**Background:**

Primary care physicians’ (PCP) referral rates to digital health programs are highly variable. This study explores whether knowledge of the digital remote blood pressure monitoring (RBPM) program and information on referral patterns influence PCPs’ intention to refer patients.

**Objective:**

This study aims to examine the relationship between PCPs’ knowledge of the digital RBPM program and information on their own prior referral rates versus their own with their peers’ referral rates and their likelihood to refer patients to the digital RBPM program.

**Methods:**

This is a mixed methods study integrating quantitative analysis of electronic health record data regarding the frequency of PCPs’ referrals of patients with hypertension to a digital health program and quantitative and qualitative analyses of survey data about PCPs’ knowledge of the program and their intention to refer patients. PCPs responded to a clinical vignette featuring an eligible patient. They were randomized to either receive their own referral rate or their own plus their peers’ referral rate. They were assessed on their intent to refer eligible future patients. Descriptive and multivariable linear regression analyses examined participant characteristics and the factors associated with their intent to refer patients. Narrative reasons for their intention to refer were thematically analyzed.

**Results:**

Of the 242 eligible PCPs invited to participate, 31% (n=70) responded to the survey. From electronic health record data, the mean referral rate of patients per PCP was 11.80% (SD 13.30%). The mean self-reported knowledge of the digital health program was 6.47 (SD 1.81). The mean likelihood of referring an eligible patient (on a scale of 0 to 10, with 0 being not at all, and 10 being definitely) based on a vignette was 8.54 (SD 2.12). The own referral data group’s mean likelihood to refer was 8.91 (SD 1.28), whereas the own plus peer prior referral data group was 8.35 (SD 2.19). Regression analyses suggested the intention to refer the vignette patient was significantly associated with their knowledge (coefficient 0.46, 95% CI 0.20-0.73; *P*<.001), whereas the intention to refer future patients was significantly associated with their intent to refer the patient in the vignette (coefficient 0.62, 95% CI 0.46-0.78; *P*<.001). No evidence of association was found on receiving own plus peer referral data compared with own referral data and intent to refer future patients (coefficient 0.23, 95% CI –0.43 to 0.89; *P*=.48).

**Conclusions:**

Physicians’ intention to refer patients to a novel digital health program can be extrapolated by examining their intention to refer an eligible patient portrayed in a vignette, which was found to be significantly influenced by their knowledge of the program. Future efforts should engage PCPs to better inform them so that more patients can benefit from the digital health program.

## Introduction

Hypertension is a leading independent and modifiable risk factor for several adverse cardiovascular outcomes including coronary artery disease, stroke, heart failure, and chronic kidney disease, as well as a wide variety of mental and metabolic conditions [1]. Long-term control of hypertension can be achieved through proven interventions including regular blood pressure (BP) monitoring [2]. Accurate BP measurements in the office can be a challenge, particularly if the patient has white coat syndrome [3].

Digital technology including digital remote blood pressure monitoring (RBPM) allows health care to conduct remote monitoring for many chronic diseases [4,5]. RBPM is expanding and shows increasing efficacy [6], cost savings, reduction in health care access disparities, reduced patient and physician burden, and ease in securing the diagnosis of white coat hypertension [7]. RBPM became more widely accepted during the COVID-19 pandemic when patients often avoided coming in for appointments [8] and health care providers were faced with a significant barrier to adequately care for their patients with hypertension and other chronic diseases. Many eligible patients with hypertension were not being seen in the office, and therefore could not be adequately or efficiently managed. Inevitably, there were also many patients developing new and sometimes uncontrolled hypertension that went unrecognized due to missed appointments. With the COVID-19 pandemic, broad legislative permissions facilitated at-home and telemedicine interventions necessary to pragmatically manage the pandemic, which led to an increase in at-home digital health care programs [9-11]. In 2020, the University of California San Diego (UCSD) implemented Project 1000 (P1000) an electronic health record (EHR)-integrated Bluetooth-enabled and team-based telemonitoring RBPM program available free for patients with uncontrolled hypertension. In this study, we explore whether knowledge of and information on referral patterns influence primary care physicians’ (PCPs’) adoption of the program through intention to refer patients to P1000.

We developed a physician survey to explore the reasons behind diverse referral patterns and analyzed the results. We hypothesize that (1) more knowledge of the digital health program is positively associated with physicians’ proclivity to refer eligible patients, and (2) information on their own prior referral rates versus their own and their peers’ referral rates could influence their likelihood to refer future patients. Using a mixed methods approach, we examined factors associated with physician intention to refer patients to P1000.

## Methods

### UCSD P1000 Program

P1000 is an innovative free EHR-integrated, Bluetooth-enabled, and team-based telemonitoring RBPM program piloted to 1000 patients whose in-office BP measured 140/90 or greater. Due to success, the program was expanded. PCPs were initially introduced to the digital RBPM program by the Population Health Services Organization (PHSO) on how to refer eligible patients. The original method of outreach was through automated phone calls, which resulted in low enrollment. An adjustment to the recruitment approach was made to involve patients’ PCPs using direct electronic referrals within our Epic (Epic Systems) EHRs system. Once the referral is entered, a digital health program team member reviews eligibility, acknowledges the referral in the EHR, and reaches out to the referred patient. Participating patients are shipped a Bluetooth-enabled digital BP cuff that automatically transmits BP data to the patient portal of the EHR via a smartphone app. Enrollees are supported by a digital health specialist via telephone or in the patient’s home for device setup and any technical issues. The devices are provided free to all enrollees and retrieved or replaced only in the case of a defective device, or if the devices are having accuracy issues and used for more than one year. The EHR system stratifies BP measures into 3 risk groups: normal, high or priority, and critical, and presents them on a daily dashboard. Nurse care managers review the dashboard and make treat-to-target adjustments per protocol, encompassing medication adjustments and behavioral change recommendations. Outcomes of the referral are documented by the team member reviewing the patient dashboard and a message is sent back to the referring provider. Since 2020, over 2500 patients have been referred to the program. BP control data analyzed since the inception of this program has shown improvement in BP control for those referred to the program regardless of participation in or continuation with the digital RBPM program [12]. However, analysis of referral patterns suggested heterogeneous referral rates among PCPs. PHSO funded the cost of the program, which remains ongoing at UCSD.

### Assessing Factors in Digital Health Adoption

Literature suggests that adoption of new primary care practices is influenced by many factors including knowledge of available resources and programs, implementation of strategies that target the needs of the PCP’s practice [13,14], and attitudes and perspectives of the clinician [7,15-17]. In particular, physician uptake of digital health care initiatives hinges upon clearly demonstrated clinical improvements for their patients, ease of integration with their clinical workflow, and easy-to-use technology that is also amply supported with technical assistance [18]. In addition, the adoption of new treatment options can be influenced by knowledge of or observation of their peer’s behavior in the case of pharmaceuticals [19], but not in other instances, for example, the uptake of EHRs [20]. Notably, PCPs are likely to be early adopters of digital health care initiatives [21]. Therefore, performance data on referral rates was deliberately included to study the influence of current performance data on the decision to refer.

We explored the reasons behind such diverse practice patterns by developing a physician survey. We had three goals: (1) assess physicians’ knowledge of the digital health program, (2) explore their proclivity to refer patients who clearly meet eligibility criteria, as portrayed in a vignette, and (3) inform them of their own prior referral rates, or their own and their peers’ referral rate, and reassess their likelihood to refer future patients.

### Design

We used an exploratory, sequential design for this mixed methods study. PCPs were recruited from Family Medicine (FM), General Internal Medicine (GIM), and Community Care (CC) at UCSD Health. Referral data for physicians in these departments were provided by an Epic analyst. Each physician was sent an individual survey link via email.

Various outreach approaches were used. FM physicians received a 10-minute presentation on the survey project and were given time to complete the survey, which was sent prior to the meeting from the FM Clinical Services Chief. GIM, Geriatrics, and CC practice physicians were informed at departmental meetings that an invitation to participate would be emailed to them and encouraged them to reply. They did not receive a formal presentation nor allotted time to answer the survey. As such, outreach to GIM, Geriatrics, and CC was less extensive.

All eligible physicians were randomized using simple randomization via a random number generator in the CRAN package of statistical analysis software “R” (R Core Team) into 1 of 2 study arms, with a link to a personalized survey that included either their own referral data (Arm A) or their own referral data plus their peers’ (own+peer) referral rates (Arm B). Two email reminders were sent to PCPs who had not responded. All PCP identifiers were kept confidential.

In the context of a quality improvement (QI) program, the initial introduction of the digital health program to PCPs in 2020 was the QI intervention. The survey in late 2023 assessed what changes they had made to their practice, what impact it had had on their practice, and what they had learned as a result of this QI intervention. Responses to the survey satisfied the requirements for maintenance of certification (MOC) credits for their boards.

**Figure 1:**
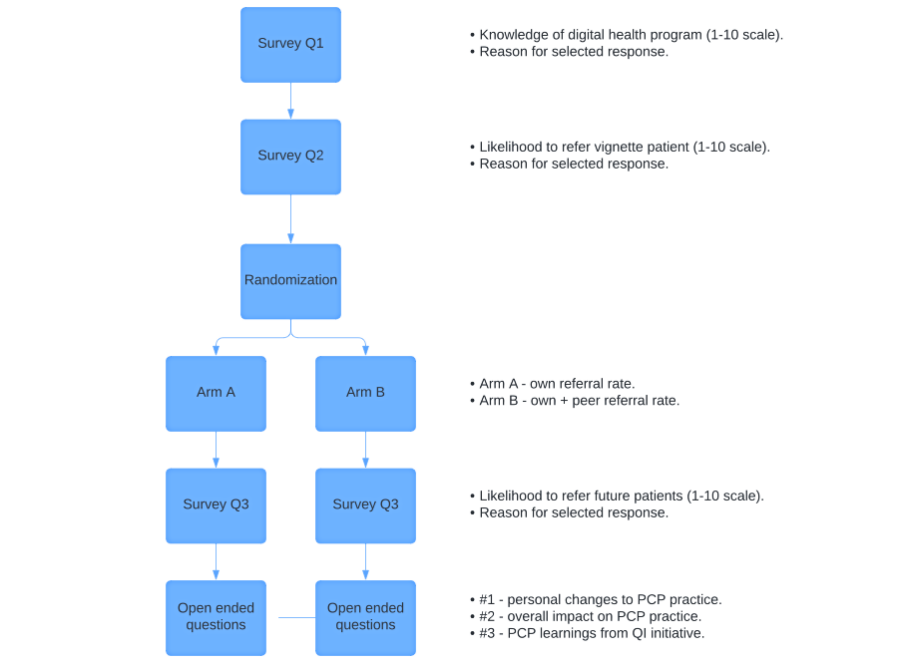
The sequence of survey questions, including at what point randomization bifurcates available data (own or own+peer) participants could view when answering survey questions. PCP: primary care physician; QI: quality improvement.

The survey contained 5 stages (Figure 1). First, PCPs rated their knowledge of the digital health program on a scale of 0 to 10 (0 being no knowledge and 10 being extremely knowledgeable) and provided a free text response explaining their score (Q1). Second, they assessed their likelihood to refer an eligible patient on a scale of 0 to 10 (0 being not at all and 10 being definitely) by their response to a clinical vignette, a valid method for distinguishing between physician practice [22]. The vignette portrayed a patient that meets the inclusion criteria of the digital health program—a 65-year-old Black male with hypertension whose BP was 150/95, and whom the provider had worked with for 2 years during which time BP remained elevated (Q2). Third, based upon random assignment, PCPs were either shown information on their own referral rate (Arm A) or shown their own and their peers’ referral rates concurrently (Arm B). The referral rates presented to respondents covered the period from October 2020 to October 2022 and were pulled from EHR data. In the fourth stage, their intention to refer future patients to the digital health program was assessed again (Q3). Fifth, qualitative reflections were gathered on what they learned, understood, and planned to change in their future practice regarding the digital RBPM program. Survey questions are included in Multimedia Appendix 1.

### Analysis

Univariate and bivariate descriptive statistics were examined. Multivariable linear regression models were used to assess factors associated with the intention to refer the patient in the vignette and the likelihood to refer future patients after seeing own versus own+peer referral rates. For the qualitative analysis, one member (RG) of the research team developed 4 codebooks (knowledge, vignette, future referral likelihood, and program takeaways) using an inductive grounded theory approach [23]. This involved iteratively refining and condensing themes extracted from a random subset of the qualitative data. In partnership with a second team member (AW), these codes were adjusted and refined. Then, these 2 research team members coded a subset of the data to ensure interpretive alignment (Cohen κ=0.84), before the remaining data were thematically coded.

### Ethical Considerations

The project was reviewed by the UCSD Aligning and Coordinating Quality Improvement, Research, and Evaluation (ACQUIRE) Committee. The ACQUIRE Committee approved this project as a QI project and its approval included a decision that the project would satisfy the requirements for maintenance of certification (MOC) credit through UCSD’s MOC Portfolio Program. Physicians who completed the survey were eligible for MOC credit. No additional compensation was provided to participants.

All PCP identifiers were kept confidential. The project manager (AW) distributed unique survey links via Qualtrics, ensuring that each physician received their individual or individual-plus-peer referral data. Survey responses were stored in a HIPAA-compliant OneDrive folder accessible only to the project manager. Prior to sharing with the research team, responses were deidentified. An identified dataset was shared solely with the Senior Health Professions Education Specialist, Accreditation and Educational Development, for the purpose of processing MOC credit. Our survey satisfied the Checklist for Reporting the Result of Internet E-Surveys (CHERRIES) [24], shown in Multimedia Appendix 2.

## Results

### Participant Characteristics and Quantitative Results

Table 1 provides an overview of the sample (N=70). The median range of years in practice was 12.5 (IQR 11-15). In total, 57% (n=40) of respondents were female, 64% (n=45) of respondents were from academic FM, 24% (n=17) of respondents were from academic GIM and Geriatrics, and 11% (n=8) of respondents were from CC (comprised of Internal Medicine or FM physicians). The average prior familiarity with the Digital Health Program was 6.47 (SD 1.81). The average likelihood of referring the patient from the vignette (on a scale of 0 to 10, with 0 being extremely unlikely and 10 being extremely likely) was 8.54 (SD 2.12). A total of 54% (n=38) of the sample received their own+peers’ referral rates, while 46% (n=32) of the sample received their own referral rates. The average referral rate of physicians from EHR data was 11.80% (SD 13.30%), while the average peer referral rate was 11.90% (SD 9.60%). The average likelihood of referring patients in the future was 8.61 (SD 1.83). Participants were randomized into 2 treatment groups—whether they were shown only their own referral rate (Arm A), or their own+peers’ referral rates (Arm B).

Table 2 compares the likelihood of referring patients within the 2 treatment groups—whether they were shown only their own referral rate, or their own and their peers’ referral rates.

Table 3 displays results from multivariable linear regression analyses to assess factors that could be associated with physicians’ likelihood to refer an eligible patient presented in the vignette (model 1) and eligible patients in the future (model 2). Explanatory variables in model 1 included length of practice (dichotomized by ≤10 years, and >10 years), PCP gender (dichotomized by female, and male or N/A or prefer not to answer), prior knowledge of the digital RBPM program, and department (reference group was FM). Prior knowledge of the digital RBPM program was significantly associated with the likelihood of referring the eligible vignette patient (coefficient 0.47; SE 0.13; 95% CI 0.20-0.73; *P*<.001).

The intention to refer future eligible patients was the dependent variable for model 2 whose explanatory variables included random assignment of information received (reference group was shown own referral rate), the self-rated likelihood to refer the vignette patient, and all explanatory variables from model 1. The likelihood of referring the vignette patient was significantly associated with their intention to refer future patients (coefficient 0.62, SE 0.08, 95% CI 0.46 to 0.78; *P*<.001).

**Table 1:** Participant demographic characteristics and department.

			Arm A—own (n=32), n (%)		Arm B—own + peer (n=38), n (%)		Total (n=70), n (%)	
**Years of practice**								
	1-5	8 (25)		14 (37)		22 (31)		
	6-10	6 (19)		5 (13)		11 (16)		
	11-20	10 (31)		8 (21)		18 (26)		
	More than 20	8 (25)		7 (18)		15 (21)		
	Prefer not to answer or N/A^a^	0 (0)		4 (11)		4 (6)		
**Sex**								
	Female	19 (59)		21 (55)		40 (57)		
	Male	11 (35)		14 (37)		25 (36)		
	Prefer not to answer or N/A	2 (6)		3 (8)		5 (7)		
**Department**								
	Family Medicine	19 (59)		26 (68)		45 (64)		
	Internal Medicine and Geriatrics	7 (22)		10 (26)		17 (24)		
	Community Care	6 (19)		2 (5)		8 (11)		

^a^Not applicable.

**Table 2:** Quantitative measures of likelihood to refer to the digital RBPM^a^ program, familiarity with the program, along with primary care physician referral rates prior to the survey, separated by a randomized arm (receiving own vs own+peer referral data).

	Arm A—own (n=32), mean (SD)	Arm B—own + peer (n=38), mean (SD)	Total (n=70), mean (SD)
Familiarity with digital RBPM program	7.09 (1.42)	5.92 (1.95)	6.47 (1.81)
Likelihood to refer patient in vignette	8.94 (1.61)	8.11 (2.45)	8.54 (2.12)
Own referral rate from EHR^b^ data	11.2% (11.6%)	12.3% (14.6%)	11.80% (13.30%)
Peer referral rate from EHR data	12.1% (10.4%)	11.8% (9.0%)	11.90% (9.60%)
Likelihood to refer patients in the future	8.91 (1.28)	8.35 (2.19)	8.61 (1.83)

^a^RBPM: remote blood pressure monitoring.

^b^EHR: electronic health record.

**Table 3:** Factors (demographics, knowledge of digital RBPM^a^ program) associated with vignette referral and likelihood to refer future eligible patients.

	Model 1^b^: likelihood to refer eligible patient in vignette					Model 2^c^: intention to refer future eligible patients			
	Coefficient	SE	*P* value	95% CI	Coefficient		SE	*P* value	95% CI
(Intercept)	6.14	1.02	<.001	4.11 to 8.18	2.21		0.93	.02	0.35 to 4.08
Practice length: >10 years (reference <10 years)	–0.28	0.54	.61	–1.36 to 0.80	0.31		0.34	.37	–0.37 to 0.99
PCP^d^ gender: male or N/A^e^ or prefer not to answer (reference: female)	–0.63	0.48	.20	–1.60 to 0.33	0.02		0.31	.96	–0.59 to 0.63
Prior knowledge of digital RBPM program	0.46	0.13	<.001	0.20 to 0.73	0.11		0.10	.26	–0.08 to 0.30
Department: CommCare (reference: Fam Med)	–1.09	0.72	.14	–2.53 to 0.36	0.48		0.47	.32	–0.46 to 1.42
Department: IntMed/Ger (reference: Fam Med)	–0.14	0.58	.82	–1.30 to 1.03	0.22		0.36	.56	–0.51 to 0.94
Shown referral rate: own+peer (reference: own)	N/A	N/A	N/A	N/A	0.23		0.33	.48	–0.43 to 0.89
Likelihood to refer patient in vignette	N/A	N/A	N/A	N/A	0.62		0.08	<.001	0.46 to 0.78

^a^RBPM: remote blood pressure monitoring.

^b^Model 1: number of observations=69; *R*^2^=0.14.

^c^Model 2: number of observations=69; *R*^2^=0.53

^d^PCP: primary care physician.

^e^Not applicable.

### Qualitative Results

After participants were asked to numerically score their knowledge of the digital RBPM program for hypertension management provided by UCSD Population Health, they were then asked to provide free text comments on their score. A total of 23 participants expressed high perceived knowledge of the program, scoring themselves 8-10 on the 10-point scale; 28 participants expressed moderate perceived knowledge (score 6-7) of the program; and 19 participants expressed low perceived knowledge (score <6) of the program. Of these, 13 participants exhibited low knowledge about the existence or basic function of the PHSO digital program, while 6 participants were aware of the program, but had low knowledge about patient eligibility criteria, which in turn meant they did not use it. Table 4 summarizes the findings.

After participants were asked to numerically score how likely they were to refer an eligible vignette patient with BP 150/95 mm Hg, they were asked to explain their reasons why. Primarily, it was clinical reasons that justified referral to the program, for example, “blood pressure is uncontrolled,” as well as the additional help provided by the program to physicians in monitoring patients of this type, for example, “Having additional oversight for this patient’s blood pressure would be beneficial especially when we as providers can only see the patient on an intermittent basis,” “[patient is] more likely to continue to engage if more people are touching base with him.” Others stated that the program is “not appropriate for all cases” and that the hypothetical patient’s trajectory also “depends on other factors.” Those who suggested they would not refer had hesitations about the program rather than clinical need; one participant said, “I forget about this resource”; while another said, “many older patients are not interested in the extra hassles and intimidated by technology.”

Physicians were randomized to be shown their referral rate to the program (Arm A) versus their own referral rate and the referral rate of their peers in the same clinic (Arm B) and asked to score the likelihood that they would refer patients to the program in the future. After giving their score, they were asked to explain the reason for their score. Nine participants (n=4 Arm A; n=5 Arm B) discussed how they already use the program and intend to continue doing so, for example, “I have been using the program more recently and have seen improvements in my hypertensive patients.” Six participants in Arm B and 2 participants in Arm A professed that their newfound understanding of the utility of the program would lead to greater referrals, for example, “Seeing this information prompts me to recognize that maybe I am not taking advantage of this resource as much as I thought.” However, some expressed hesitations about future referrals. Notably, 9 participants in Arm A and 4 participants in Arm B hesitated about further patient adoption of the program, worrying about technological aptitude: “I find most of my patients do not enroll in the program because they cannot manage the technology involved”; about insurance coverage: “I don’t know about insurance coverage for this service”; or simply refusing: “most patients referred decline.” Five participants from Arm A and 1 participant from Arm B had no intention to refer.

**Table 4:** Quantitative score of perceived knowledge of the digital program (0-10) alongside qualitative responses.

Knowledge of the digital RBPM^a^ program and code, and quote	
**Very knowledgeable (8-10), n=23**	
	“Used the program frequently”
	“[I] Feel confident in how to access their services, how to view the home blood pressures, and how to communicate with the main nurses, pharmacists ... etc.”
**Somewhat knowledgeable (6-7), n=28**	
	“Refer many patients, see some messages, but not truly aware on logistics and options of services provided”
**Lacking knowledge of program features (<6), n=13**	
	“I have no idea on how this program works and how the data collected help me directly manage my patients. Or whether the Population health will manage these patients for me.”
**Lacking knowledge of patient eligibility (<6), n=6**	
	“Unclear which patients are eligible.”

^a^RBPM: remote blood pressure monitoring.

To meet the requirements for MOC, participants were asked to reflect on the QI intervention, and how it would affect their future practice. They were asked about what they would change personally and clinically because of the intervention, as well as what their key learnings and takeaways were. Participants were excited by how the clinical data would improve their practice and patient gaps. Some simply appreciated having more, and more continuous, data: “I have gained valuable data about my patients from their home monitoring, and have been able to, and felt more comfortable to intervene with medical treatment when needed for blood pressure that is uncontrolled.” Many highlighted how at-home monitoring clearly demonstrated the extent of overdiagnosis of hypertension stemming from white coat syndrome: “I also found that patients with anxiety and white coat hypertension were less likely to be overtreated with medications when referred to this program.” Having contemporaneous data would lead to better clinical outcomes, many felt: “This blood pressure monitoring program and hypertension management program can lead to better cardiovascular outcomes and better overall health for patients because of the real-time monitoring.”

A total of 29 (41%) providers expressed a determination to refer to PHSO more often as part of their clinical practice, acknowledging its value in comments, including the following: “I plan to utilize the PHSO HTN program more often. Also to use it when indicated. This improves patient’s outcomes.” Some went further, noting the availability of collaborative programs within UCSD Health that may improve clinical outcomes and reduce clinician workload: “Better understanding of health systems programs that can address complex cardiovascular care from the primary care perspective.” A few noted that the program brought them confidence and assurance: “Participating in the program reassured me about the quality and accuracy of their reading.”

Another theme emerging from the reflections was that providers were happy with how the patients were empowered to participate in their own care through the program. One said that patients “were excited when starting digital health monitoring,” while another said, “I have learned the benefit of patient participation in collecting data to view where they are in their health.” One went further, saying “patients can be more engaged in their health when we make access to decision-making easier.” A patient education theme also emerged: “The digital health options have increased patient compliance, participation, and knowledge in the management of hypertension.”

Finally, more physicians in Arm B (n=10) than in Arm A (n=6) expressed an increased desire and capacity to harness medical technology in the future, particularly in concert with their patients, due to the QI intervention. One provider suggested they underestimated the ability of their older people, nonnative speaking patient population to do this: “Maybe not underestimating PHSO ability to provide services to my elderly Mandarin-speaking population through the use of interpreters as many of them are still tech-savvy enough to use smartphones so could likely incorporate similar technology at home.” By using technology, patients and providers could be in closer contact, but also better connected to the wider medical infrastructure for a more thorough, networked care experience: “It renewed a sense of institutional support for keeping our patients healthy. It also renewed my appreciation that there are teams of health care staff in our system who stand ready to assist us in our work and keep us focused.” Finally, the program enabled physicians to practice state-of-the-art medicine: “I feel like the QI project has made my practice more up to date with current medical care trends.”

Table 5 summarizes key facilitators and barriers to physician participation in the program. There are 4 key facilitators to ongoing physician participation in the program. First, getting an initial referral underway is important, as the benefits of the program become apparent quickly and diffuse many initial apprehensions. Second, having BP data at more regular intervals provides greater data clarity to inform clinical decision-making, and identifies instances of white coat hypertension. Third, physicians are encouraged to continue in the program by 2 improvements to patients; first, their clinical care, but also the education, empowerment, and agency they receive by participating in their care in collaboration with their doctor. Fourth, physicians can benefit from advances in medical technology and are well supported by a team while doing so. Four key barriers to physician participation in the program also surfaced. First, PCPs express concerns about patients’ ability to adhere to the program, like an inability to use the technology or reluctance to take BP measurements. Second, some PCPs view the program as reducing their control over managing their patient’s hypertension, and possibly increasing their workload in the process rather than reducing it. Third, some physicians do not understand the program structure, thinking it costs patients or requires insurance. Fourth, physicians were unclear about patient eligibility to enroll in the program.

**Table 5:** Qualitative categories of facilitators and barriers to physician use of the digital RBPM^a^ program.

Subtheme		Quote
**Facilitators to PCP^b^ participation in the program**		
	Getting PCPs started is vital, once they begin using the program they see improvements in patients and understand it better	“I discovered the benefits of home BP readings which directed to manage and control my patients’ Hypertension. But, I was not sure that their readings were accurate or the quality of their devices since they were not calibrated. Participating in the program reassured me about the quality and accuracy of their reading.”
	Continuous clinical oversight improves PCP understanding of hypertension, reduces white coat and other clinical errors	“I also found that patients with anxiety and white coat hypertension were less likely to be overtreated with medications when referred to this program”
	Observing 2-fold patient benefit, whereby patient care is improved but also patient experience and agency is heightened	“I have learned the benefit of patient participation in collecting data to view where they are in their health”
	Program is a way to harness advancing medical technology and increase patient touchpoints with team-based support	“I’m learning about the power of harnessing remote digital technology to provide another layer of support to patients outside of the office. The more regular the medical contact with a poorly controlled medical problem the better.”
**Barriers to PCP participation in the program**		
	Concerns about patient capacity to adhere to the program	“Many older patients are not interested in the extra hassles and intimidated by technology”
	Fear over losing control over how patient hypertension is managed	“I feel like it is taking away from my own work for helping patients with their HTN”
	Misunderstanding of program funding structure	“I don’t know about insurance coverage for this service”
	Misunderstanding of eligibility criteria for the program	“I was not aware who qualifies for the program”

^a^RBPM: remote blood pressure monitoring.

^b^PCP: primary care physician.

## Discussion

### Principal Results

Quantitative analyses suggest knowledge of the program was significantly associated with physicians’ likelihood to refer the eligible vignette patient, which itself was significantly associated with the intention to refer future patients. Those with high knowledge scores frequently explained how this knowledge came from frequent use and experience with the program, and those with low knowledge scores frequently expressed reservations about basic eligibility criteria and the clinical and practical utility of the program, which indicates lower use.

Similarly, those more inclined to refer the vignette patient tended to mention their experience receiving additional help referring prior patients with these clinical needs and were less likely to be concerned about whether their patients could use the technology—other studies indicate fears about patients using digital RBPM are often overestimated by physicians [9]. Some of those who were reluctant to refer the vignette patients expressed the need for more clinical context, which, in essence, is what the program offers with minimal additional clinical or administrative burden to PCPs. Additionally, as seen in existing literature [15], some providers are hesitant to refer as they feel it affects the clinician’s ownership of the patient’s care.

Despite some participants identifying barriers to referring patients, reflections from PCPs about the program were generally positive. Many referenced the improved clinical outcome it produced, and its capacity to provide patients with education and enhanced agency over their care as key reasons why they would use the program in the future. One important clinical insight, that supports other studies of digital monitoring for hypertension [25], is that the program helps clarify the incidence of white coat syndrome—one participant said “I also found that patients with anxiety and white coat hypertension were less likely to be overtreated with medications when referred to this program”—which wastes clinical resources, as well as physician’s and patient’s time [3]. One previous study discovered as many as 8% of their cohort had white coat syndrome [25] when enrolled in a digital RBPM program; our qualitative data gently supports this clinical justification for digital RBPM programs.

Criteria for hypertension can vary in different clinical contexts, and misalignment between being categorized as hypertensive in a PCP native data environment (>130/80 mm Hg) and being a candidate for the population health program (>140/90 mm Hg) may have left some physicians questioning the appropriate time to refer. How to best prompt physicians to consider this resource for eligible patients remains a somewhat unanswered question. In addition to PCP awareness of clinical eligibility criteria for the program—which qualitative and quantitative data suggests is mixed—it is important for physicians to be aware of the program’s payor-neutral status. Patients face no cost-sharing in this program. Some PCPs incorrectly believed that insurance status and financial limitations might preclude patient participation.

Furthermore, it is vital that health care organizations ensure that team-based services like these are perceived as supportive partners rather than adding to already-excessive administrative burden. In the future, it would be prudent to solicit physician input on determining the most appropriate channels for communication regarding patients comanaged by the PHSO digital health program and PCP. This applies to communicating eligibility, enrollment, and ongoing monitoring. Several physicians who used the program frequently noted that it not only supported and empowered patients, but also actively reduced physician burden, particularly with patients who had poor access to consistent health care for reasons of socioeconomics, geography, or age-related mobility issues. Simple BP follow-up appointments can easily clog a physician’s already limited availability, especially if no-shows are frequent, while also placing demands on patients ill-suited to frequent clinic visits due to chronic disease burden. A collaborative emphasis that does not place responsibility solely on the PCP is most appropriate [9].

Some physicians reported concerns regarding patient receptivity to digital monitoring tools, whether it be older patients who are less technologically savvy, patients who are non-English speakers, or those with limited financial resources—highlighting the need to address these potential barriers more proactively to enhance the footprint of this program to those in need. There may be patients with negative attitudes about having others involved in their care, and perhaps that perception reflects PCP practice habits if physician autonomy in care management tends to be prioritized or viewed as superior to referring to other members of the health care team. Though one might intuit that earlier career physicians may be more receptive to team-based care initiatives such as this program as a new way of practice, our data did not show evidence of an effect in the likelihood to refer across years of practice groups. Furthermore, physician concerns regarding their autonomy in patient management did arise in qualitative findings, highlighting the need to emphasize the collaborative nature of such initiatives which recognizes the physician as an essential member of a patient’s care team.

This study found no evidence of an effect of seeing own+peer’s referral rates compared with seeing only their own referral rate. That said, it is unclear whether awareness of the clinical practices of one’s peers might warrant an immediate impact on one’s own practices but over time could be a factor of influence. As such, future studies might follow a cohort for a longer time, sharing peer referral data rates, to determine if the effect has a greater influence over time.

### Limitations

Outreach approaches varied. FM physicians received a 10-minute presentation about the survey project and were given time to complete the survey, which had been sent prior to the meeting via an email invitation from the FM Clinical Services Chief. GIM, Geriatrics, and CC practice physicians did not receive a formal presentation nor allotted time to complete the survey, which may have contributed to the underrepresentation of these 2 practices.

Patient referrals were attributed to the patient’s PCP in the EHR. Patients may not see their attributed PCP at every visit, so a colleague, Resident Physician, or Advanced Practice Provider may have made the referral. Thus, the referral rates as abstracted might not be entirely representative of any given physician’s actual personal utilization of the program based upon the data, though we presume that this general foundation of comanaging patients in academic practices likely evens out such that it still is an adequate approximation of their referral patterns. Given our sample size is relatively small and collected at a single institution, this study may be underpowered and limited in its generalizability. Future studies might seek larger PCP populations across multiple institutions situated in diverse socioeconomic and geographic contexts with variable overall chronic disease burden. They might also investigate whether differences exist between FM, Internal Medicine, and other departments, or between academic and community settings, in the acceptance and utilization of digital health for chronic disease care. Finally, the program at the time of the study was limited to English-speaking patients only, limiting the potential patient pool eligible for the study.

### Conclusions

This study indicates that PCP knowledge and understanding of digital health monitoring programs, in terms of clinical outcome, practical application, and the reduction of work burden, is crucial to increased use and thus widespread effectiveness of these programs. Contextualizing PCP referrals with their peers’ referrals showed no evidence of an effect in furthering their intention to adopt the program. Since remote patient monitoring has an increasing volume of empirical support for improving clinical outcomes, we encourage more robust communication of the effectiveness of these programs, their ease of use, and the availability of support to PCPs to increase enrollment and improve population health.

## Appendix

Multimedia Appendix 1 Multimedia survey supplement.

Multimedia Appendix 2 CHERRIES framework.

Multimedia Appendix 3 Deidentified data.

## Abbreviations

BP: blood pressure
CC: Community Care
CHERRIES: Checklist for Reporting the Result of Internet E-Surveys
EHR: electronic health record
FM: Family Medicine
GIM: General Internal Medicine
MOC: maintenance of certification
PCP: primary care physician
PHSO: Population Health Services Organization
QI: quality improvement
RBPM: remote blood pressure monitoring
UCSD: University of California San Diego

## Data Availability

All data generated or analyzed during this study are included in this published article under Multimedia Appendix 3.
