# Supplementary material for: Factors Influencing Primary Care Physicians’ Intent to Refer Patients With Hypertension to a Digital Remote Blood Pressure Monitoring Program: Mixed Methods Study
Source: J Med Internet Res. 2025 Mar 24;27:e64933. doi: 10.2196/64933 (PMC11976174; doi:10.2196/64933)
Supplement: Multimedia Appendix 1 [file jmir_v27i1e64933_app1.docx]

**1a.** How would you rate your knowledge of the digital health program for hypertension management provided by UC San Diego Health Population Health Services Organization (PHSO) on a scale of 0-10? (0 = not at all, 10 = know everything about it)

**1b.** Please explain the reason you chose this number:

**2a.** Suppose you have a 65-year old, Black, male patient with hypertension whose average BP was 150/95 over the last two years. You have been working with him for the last year and his blood pressure remains high. On a scale of 0-10, how likely are you to refer patients like him with hypertension to PHSO’s Digital Health Program? (0 = not at all, 10 = definitely)

**2b.** Please explain the reason you chose this number:

**3a.** After seeing your information on referrals to PHSO’s digital health program, on a scale of 0-10, how likely are you to refer patients with unmanaged hypertension to PHSO’s digital health program in the future? (0 = not at all, 10 = definitely)

**3b.** Please explain the reason you chose this number:

**4a.** CHANGE: In 3-8 sentences, describe what changes you personally made in your practice during this QI effort.

**4b.** IMPACT: In 3-8 sentences, please describe how your practice has been impacted since you completed your participation in this QI effort.

**4c.** LEARNING: In 3-8 sentences, please describe what you learned as part of your participation in this QI effort
